# New exploration of creativity: Cross-validation analysis of the factors influencing multiteam digital creativity in the transition phase

**DOI:** 10.3389/fpsyg.2023.1102085

**Published:** 2023-02-24

**Authors:** Weilong Chen, Baohua Wang, Yi Chen, Jing Zhang, Yuchun Xiao

**Affiliations:** ^1^Zhejiang Institute of Social Governance and Communication Innovation, Communication University of Zhejiang, Hangzhou, China; ^2^School of Business Administration, Zhejiang Gongshang University, Hangzhou, China

**Keywords:** cross-validation analysis, digital creativity, influencing factors, MTS theory, transition phase

## Abstract

Multiteam digital creativity (MTDC) is a new domain of creativity study that fits the new developments of the digital era, thus scholars have called for exploring MTDC in the fine-graining phase. This paper responds to this call, and adopts two studies and cross-validation analysis to explore the theoretical framework of the impact factors of MTDC in the transition phase. Study 1 adopts the qualitative analysis method of rooted theory to explore a more comprehensive impact factor and to maximize the new theory’s saturation. Study 2 adopts the CL-WG DEMATEL method, one analysis method of group decision-making and optimized concept lattice, which could cross-validation analyze the results of Study 1 and further determine the importance of the factors. The results of the studies indicate that the influencing factors of MTDC are multilevel, and the factors such as the organizational digital climate, team psychological empowerment, individual digital cognition and emotion, and leadership competence have greater impacts on MTDC. This indicates that the transition phase has a unique internal mechanism. This paper constructs a theoretical framework of factors influencing MTDC in the transition phase and provides new theoretical and practical references for how organizations could fully stimulate MTDC in the digital era. In addition, the cross-validated analytical method further enriches the study tools in the domain of organizational behavior.

## Introduction

Digital technology has led to significant changes in the organizational environment and brought us into the digital era (Yoo et al., 2010; Grover et al., 2022). Digital technology differs from previous virtual network technologies in its transferability, flexibility and editability, most organizations are eager to embrace digitalization to gain a new competitive advantage (Shao et al., 2021). Creativity is also one of the most needed competitiveness in the digital era (Van Rensburg et al., 2021). Digital creativity, a new cross-domain of digital technology and creativity, is important competitiveness in the digital age (Lee and Chen, 2015). Digital creativity refers to various forms of creativity based on the digital foundation or driven by digital technology (Shao et al., 2021; Van Rensburg et al., 2021). However, the study on digital creativity in the domain of organization is relatively limited, so some scholars call for more relevant research (Shao et al., 2021; Zhang et al., 2022).

The iterative development of digital technology and knowledge further drives the prevalence of the multiteam working model (Rapp and Mathieu, 2019; van de Brake et al., 2020). Multiteam working models can enhance an organization’s ability to innovate and adapt effectively to dynamic and complex organizational environments (De Vries et al., 2016; Luciano et al., 2020). Thus, scholars have called for more attention to organizational issues from a multi-team perspective (Lei et al., 2022; Wu et al., 2022). The multiteam system (MTS) theory provides a reference theory for organizations’ multiteam working models. MTS is an interdependent system of multiple teams interacting to achieve a set of subgoals guided by a common goal (Mathieu et al., 2001), which has gradually become a hot topic (Luciano et al., 2018; Zaccaro et al., 2020). Based on the above digital creativity and MTS theory, multiteam digital creativity (MTDC) is defined as the generation of novel and practical ideas, services, and processes, driven by digital technologies or digital environments, which is a hybrid of multi-level creativity synergy (Zhang et al., 2022).

MTS theory proposes that the multiteam collaboration process comprises the transition phase and action phase, which is called the performance episode (Marks et al., 2001; Mathieu and Schulze, 2006). The transition phase focuses on multiteam evaluation and planning activities, including task analysis, goal setting and strategy development, to guide the teams toward multiteam goals; and the action phase focuses on multiteam goals and systematic monitoring, mutual support among teams, shared feedback and mutual synergy to ensure the achievement of organizational goals. Different phases have unique characteristics (Torres et al., 2021). Thus, targeted research should be adopted for different phases. This paper focuses on the transition phase. Specifically, this paper explores the influencing factors of multiteam digital creativity in the transition phase.

Previous studies on factors influencing digital creativity have been piecemeal. E.g., the exploration of creativity influencing factors based on virtual design teams (Chamakiotis et al., 2013), the study of digital creativity themes and framework based on literature compilation (Lee and Chen, 2015). The model of factors influencing MTDC in the transition phase is a new domain. Thus, this paper adopts two interactive validated exploratory research methods to enhance the reliability of theoretical exploration. Study 1 adopts the qualitative analysis method-rooted theory. This method is capable of effectively discovering the impact factors of new theories through multilevel coding. Therefore, this is an exploratory method to explore a more comprehensive impact factor and to maximize the saturation of new theories (Hoda et al., 2012). Study 2 adopts the conceptual lattice-weighted group DEMATEL analysis method (CL-WG DEMATEL method), which is a quantitative measure that considers the opinions of the expert group, the proportion of expert weights and the degree of interaction between influencing factors (Shi et al., 2016). This method uses group decision-making and optimized concept lattice, which allows cross-validation of the results of Study 1 and further analysis of the importance of the impact factors. Thus, this is an effective research method to further increase the credibility of the new theory exploration. Both methods are exploratory tools based on a cognitive perspective, which can be cross-verified, and is confirmed to make more comprehensive and accurate decisions on influencing factors (Xiao and Zhang, 2020; Zhang et al., 2022).

Overall, this paper has some contributions. First, this study constructs a new theoretical framework of the impact factors of MTDC in the transition phase, which provides future research for digital creativity, and a practical guide for organizations to stimulate digital creativity. Second, this paper adopts two cross-validated research methods to explore the new theoretical framework, which further enriches the research tools in the field of the organization. Third, this paper studies MTDC specifically for the transition phase, further fine-graining MTS theoretical research while making it easier for organizations to provide more targeted practical references for the special phase. Finally, through the CL-WG DEMATEL method, this paper finds that the factors affecting MTDC are unique, and the importance of various factors is different. In addition, this paper responds to the theoretical call and enriches MTS and creativity theory.

## Literature review

### Multiteam system theory

The multiteam system refers to a system consisting of two or more teams interacting to achieve a set of goals. In such a system, the subteams within the system have their own proximal goals but interact under a common vision; and there is interdependence between the subteams, at least in terms of inputs, processes or outputs (Mathieu Mathieu et al., 2001). MTS theory is a development of the team behavior process theory (Marks et al., 2001). MTS theory states that the multiteam behavior process is similar to the team process and includes two different behavior phases, called the transition phase and the action phase (Mathieu and Schulze, 2006). As in the introduction above, the two phases have different concerns. Therefore, it would be meaningful to focus on one specific phase (Torres et al., 2021). This paper responds to the theoretical call and focuses only on the transition phase to further fine-graining MTS theoretical research.

More and more organizations are focusing on multiteam working models, and scholars are also paying more attention to MTS theory from multiple perspectives, such as multiteam collaboration (Davison et al., 2012), leadership (DeChurch and Marks, 2006), cross-border identity (Cuijpers et al., 2016), behavioral processes (Van den Berg et al., 2014), and cognition and motivation (Lanaj et al., 2013). An experimental study found that the coordinated actions developed by unit team boundary managers and system leaders had a positive impact on outcomes only when collaboration revolved around the team’s most critical tasks (Davison et al., 2012). One study found that multiteam identity influences multiteam output after mediation through team conflict (Cuijpers et al., 2016). Furthermore, the social identity of multiteam systems will have negative effects in task complexity situations due to more individual dissipation (Porck et al., 2019). One overview stated that important influences on multiteam effectiveness include coordination processes; leadership structure; cognitive, affective, and motivational emergent states; MTS boundary states (internal or external); and team variability (geographic, functional, cultural, and normative; Zaccaro et al., 2020). One paper proposed that diversity and dynamism were the two key structural features of MTS; and the key factors influencing MTS outcomes included belonging needs, cognitive abilities, and affective states (Luciano et al., 2018). Overall, the factors influencing the output of multiteam are diverse and complex.

### Creativity and digital creativity

Creativity is defined as the generation of ideas, products, processes, and services that are novel and useful to individuals or teams (Amabile et al., 1996). Creativity also is the result of the interaction between an individual and the organizational environment (Woodman et al., 1993). From a systematic view, creativity includes the “4Ps” elements, called the individual creator (person), the creative process (process), the individual creator (person), the creative process (process), the creative product (product), and the creative environment (place; Runco and Kim, 2013).

In the digital era, there are increasingly more “digital natives,” and digital innovation is a hot topic. However, there is relatively little research on digital creativity. Some previous studies explored creativity based on an Internet perspective, such as focusing on the factors influencing creativity in virtual design teams (Chamakiotis et al., 2013), and exploring the incentives and impediments to creativity in virtual teams based on rooting theory (Ocker, 2005). However, digital technology is both similar to and different from the earlier networked virtuality. As stated above, digital technologies possess transferability, flexibility and editability (Shao et al., 2021).

Digital creativity is a new way to explore creativity and showcase creativity using digital tools and technologies, is becoming an important skill in the workplace(Van Rensburg et al., 2021), and is an important part of people’s daily lives in the digital era; thus, organizations should pay high attention to digital creativity (Pérez-Fuentes et al., 2019). Digital creativity is creativity inspired by the use of digital tools and technologies (Shao et al., 2021; Van Rensburg et al., 2021). Lee and Chen (2015) define digital creativity as the diverse creativity of an individual, team or organization driven by digital technology and the ability of people to demonstrate novelty and usefulness in a variety of innovative activities using digital technology or through digital technology products. According to the literature review, digital creativity is a special form of creativity that emphasizes the formation of novel and practical ideas based on a digital environment or driven by digital technology (Amabile et al., 1996; Lee and Chen, 2015; Van Rensburg et al., 2021).

### Multiteam digital creativity and influencing factors

From the MTS perspective, the study of MTDC is a new paradigm in creativity theory research. Woodman et al. (1993) proposed the theory of creativity interactions among individuals, teams, and organizations that provides a hybrid cross-level perspective on creativity theory. Drawing on MTS theory and creativity theory, this paper proposes the concept of MTDC. Unlike traditional creativity, MTDC has three distinctive features: (1) it is an interdependent multiteam system that interacts to achieve a set of subgoals guided by a common vision, whereas creativity is the result of hybrid interactions and is more systematic, dynamic, and complex; (2) it emphasizes a digital environment or is driven by digital technology; and (3) it is novel and practical in the formation of ideas (Amabile et al., 1996; Zhang et al., 2022).

The factors influencing creativity are diverse and complex. The creativity component model proposes that the three important structural elements of creativity are the following: expertise, creative skills, and intrinsic motivation (Amabile, 1988). The interaction model of creativity suggests that creativity is the result of the interaction of multilevel factors such as individual cognition and emotion, team norms and team motivation, organizational culture and organizational resources, and other factors (Woodman et al., 1993). This multi-level research framework provides good inspiration for the follow-up study of influencing factors.

Studies on the factors influencing digital creativity are relatively limited. Research through rooting theory has found that significant enhancers of creativity in virtual teams include the existence of a variety of social influences, a collaborative team climate, stimulating colleagues, etc. (Ocker, 2005). Inhibiting factors that affect creativity in virtual teams include technical difficulties, lack of shared understanding, time pressure, domain knowledge, etc. (Ocker, 2005). E-leadership, trust, subgrouping, conflict and diversity may be the key to solving the problem (Chamakiotis et al., 2013). Through empirical studies, some scholars have confirmed that digital creativity is positively correlated with digital inclination, the digital environment, and the professional field (Lee D. S. et al., 2013), also influenced by authentic leadership, sharing team climate, psychological empowerment, and information sharing (Hahm, 2017). Others find that technology digital affordance, digital knowledge, and task variety affect digital creativity (Shao et al., 2021). The literature review also revealed that factors influencing digital creativity include human, technological, environmental, creative arts, learning, and policy (Lee and Chen, 2015). Some scholars have also proposed that the factors influencing digital creativity are multi-layered, including individuals, teams, and organizations (Lee D. S. et al., 2013). Multi-level influencing factors may also include individuals, technology, teams, and organizations (Chamakiotis et al., 2013). Zhang et al. (2022) initially explored the influencing factors of MTDC in the action phase, and further called for a deeper exploration of MTDC influencing factors from the transition phase.

In general, there have been more studies on individual or team creativity, and relatively few on digital creativity. However, research on digital creativity from an MTS perspective is limited. Multiple teams are an important and common organizational structure (Mell et al., 2020; Zaccaro et al., 2020). To bridge this gap, this paper explores the factors influencing MTDC in the transition phase from the perspective of MTS theory. Two studies engaging in cross-validation exploratory research, each using a different method (Study 1 and 2), systematically investigate the frame of the factors influencing MTDC in the transition phase.

## Study 1: Identification of the influencing factors

### Method

Study 1 adopts the qualitative analysis method, which focuses on conceptualization and theory scope (Urquhart et al., 2010), which can effectively uncover new influences on the theory (Mello and Flint, 2009; Hoda et al., 2012). This study adopts video or face-to-face interviews and questionnaires. The process of selecting interviewees is based mainly on the matching principle to ensure sample representativeness and theoretical saturation (Zhang et al., 2022).

### Participants and procedure

To increase the representativeness of the semistructured interviewees, this paper adopted three main steps. First, 15 managers from four high-tech enterprises in which digital technology had been deeply embedded were selected as the preliminary interviewees, then asked to recommend additional 55 multiteam managers. They have a more in-depth understanding of MTDC.

Second, based on the definition of MTS and the transition phase in the performance episode (Mathieu and Schulze, 2006), investigators fully communicated with the interviewees about the relevant concepts so they can deeply understand the research semantics of MTS and identify operational definitions and fitness criteria. First, the selected object should be to manage two or more different teams with one or more common goals, and each team has strong interdependence in at least one aspect, which conforms to the characteristics of the multiteam. In addition, in the transition phase, the core control standard is the preparation of at least one multiteam common plan and the assignment of tasks to two or more teams.

Finally, based on the preliminary interview results, 35 managers who met the criteria were selected for at least 1 h of interviews. The basic information of the interviewees shows in Table 1. The following topics were interviewed. What are the multiteam composition structures and characteristics? How does digital creativity arise? What is the specific performance of the company in terms of digital creativity or digital innovation in the transition phase? What factors generate or hinder digital creativity? Discuss the specific performance of MTDC in your organization and other aspects.

**Table 1:** The basic information of the interviewees.

Category	Content	Frequency	Percentage (%)
Gender	Male	22	62.86
	Female	13	37.14
Education	Bachelor’s or below	16	45.71
	Graduate or above	19	54.29
Age	35 or below	21	60
	35 or above	14	40

### Analysis

According to the procedure, the coding includes excerpting, coding, and categorizing, then forms three levels of coding based on the analysis and categorization (Glaser, 1978).

#### First-level coding

Based on the data collected, three study team members completed the excerpting, coding, and categorizing. To improve the effectiveness of coding, the team leader first conducted a trial coding and proposed a coding specification, and the other 2 researchers coded separately according to the same specification and checked each other’s coding consistency. In addition, those concepts that reached a consensus were put into the initial concept base while those concepts that did not reach a consensus concept were decided collectively. Interviews, questionnaires, and coding were performed in parallel individually.

Then, based on the next batch of interviews or questionnaires, invalid concepts were eliminated and clustered to form valid concepts. Statistics were conducted based on the coding, and the valid concepts with the highest mention frequency were screened out and finally summarized into valid codes. Some representative interviews were organized into a coding library, as shown in Table 2.

**Table 2:** Coding database of some interview contents in the transition phase.

Effective Concepts	Frequency	Overview of some of the interviews
Digital Cognition	22	Can understand and grasp digital development well and have a clear understanding of the target tasks. Good cognition of multiteam needs
Job Embedding	15	In a multiteam system, interdependence is important. Everyone needs to be actively engaged in their work and willing to sacrifice themselves
Positive Emotions	18	The individual is enthusiastic, positive, active, alert, full of energy, and able to engage happily in their work
Network Resources	26	Rich social resources and access to novel and useful information from multiple sources, especially digital innovation resources
Cross-Border Capabilities	12	Leaders are able to coordinate well across multiple teams, especially between digital technology teams and traditional teams
Management Skills	16	Ability to choose the right leadership style to manage and motivate everyone
Self-Motivation	12	Having a certain time and space allows individuals to combine work and interests to explore new things
Sense of Responsibility	24	Have a strong sense of responsibility and strive to find ways to identify and solve problems
Team Belonging	17	Identify very much with your team, individuals are an important part of the team, and team goals are very meaningful
Task Conflict	19	When setting goals, assigning tasks, or analyzing problems, the group sometimes heatedly discusses and sometimes argues
Relationship Conflict	22	There are conflicts in emotional relationships and do not see eye to eye with each other
Process Conflict	10	Disagreement on the way to work and the process
Diverse Structures	21	Different teams possess their own specialized skills, digital competencies and knowledge structures. Better able to adapt to digital
Open Structure	13	The team is open to dynamic change. There are multiple participating subjects
Digital Structure	11	Having digital high-tech experience in top companies will bring us a more advanced perspective
Shared Goals	8	Have one or more of the same goals
Task Interdependence	15	The tasks of the various teams are interconnected, with the digital team being particularly important
Multiteam Trust	17	The teams trust each other and can work well together
Digital Innovation Culture	28	The organization advocates digital innovation, everyone includes each other, and there is a special emphasis on digital development
Motivational Mechanism	15	With moderate motivational mechanisms, special attention is given to market needs in the digital field
Digital Training	17	Proactively organize digital training and actively guide digital innovation
Digital Infrastructure Resources	14	Digital infrastructure is an important support for organizing digital innovation
Digital Human Capital	20	The organization is able to provide strong support when people with all skills, especially digital expertise, are needed
Platform and Ecological Resources	15	Matching digital platforms and ecosystems are very important. If you do not proactively integrate you will miss the windfall of the moment

#### Second-level coding

This process focused on reclustering the valid concepts formed in the first-level codes. First, the study team clustered the valid concepts into different categories to form a preliminary second-level coding library.

Second, six experts in organizational behavior and human resources were invited to analyze the codes. Through interview induction, theoretical reference, and expert opinions, the codes were clustered into eight primary categories. Table 3 shows some concepts of the second-level codes.

**Table 3:** Main category library of the second-level coding in the transition phase.

Main Categories	Effective Concepts	Main Category Connotations
Digital Cognition and Emotion	Digital Cognition	Have a clear understanding of the organization’s digital strategic goals and be fully engaged in their work
	Job Embedding	
	Positive Emotions	
Leadership Competence	Network Resources	Leadership’s management style, resources and capabilities promote creativity
	Cross-Border Capabilities	
	Management Skills	
Team Conflict	Task Conflict	A perceptual process arising from differences or dissonance in goals, perceptions, and visions among team members, classified as task conflict (TC), relationship conflict (RC), and process conflict (PC; Jehn and Mannix, 2001)
	Relationship Conflict	
	Process Conflict	
Team Psychological Empowerment	Self-Motivation	The psychological perception of being empowered, as experienced collectively by team members, is an intrinsic and continuous work motivator that enhances an organization’s performance (Rosen, 1999; Chen et al., 2022)
	Sense of Responsibility	
	Team Belonging	
Multiteam Structure	Diverse Structures	The multiteam components, including the structures of diversity, openness and dynamic adaptability
	Open Structure	
	Digital Structure	
Multi-Team Orientation	Shared Goals	Guided by shared goals and task interdependence, teams relate to each other with trust and eventually develop a complementary multiteam orientation (Wijnmaalen et al., 2019)
	Task Interdependence	
	Multi-Team Trust	
Organizational Digital Climate	Digital Innovation Culture	Multiteam members’ perception of an organization’s supportive climate and the organization’s stimulation of members’ creativity
	Motivational Mechanism	
	Digital Training	
Digital Resource Matching	Digital Infrastructure Resources	The digital infrastructure, digital platforms, and ecosystems that are available in the organization are the foundation. The combination with human resources is the guarantee of digital creativity
	Digital Human Capital	
	Platform and Ecological Resources	

#### Third-level coding

With reference to creativity theory and MTS theory, a theoretical structure frame was established by logically analyzing the 8 s-level master categories. The four key categories of MTDC in the transition phase were mined according to the hierarchical structure at the individual, team, multiteam, and organizational levels.

### Results

**Figure 1:**
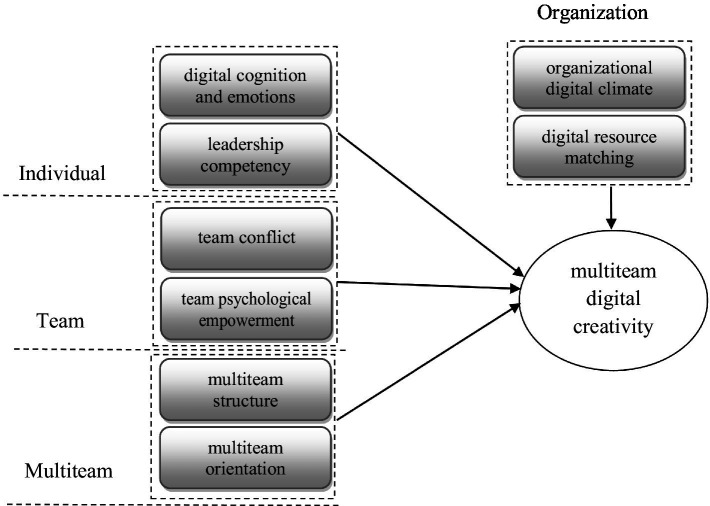
Frame of the factors influencing MTDC in the transition phase.

Based on the results of the above three-level coding and the MTS theory (Mathieu et al., 2001), this study finds that the frame of the factors influencing MTDC in the transition phase has four levels, specifically the individual level (digital cognition and emotions, leadership competency), team level (team psychological empowerment and team conflict), multiteam level (multiteam structure and multiteam orientation), and organizational level (organizational digital climate and digital resource matching). The four levels of interaction influence the process and outcome of MTDC. As mentioned earlier, the transition phase of the multiteam behavior process has its uniqueness. The frame shows in Figure 1.

Based on the frequency aggregation in Table 1 and the main category in Table 2, this paper finds that the individual level’s digital cognition and emotion, leadership competence, team psychological empowerment, and organizational digital climate have greater impacts on MTDC in the transition phase.

## Study 2: Validation and centrality analysis

### Method

This section adopts the optimized DEMATEL analysis method-the conceptual lattice-weighted group DEMATEL method (CL-WG DEMATEL method). The DEMATEL method has been widely used to identify and evaluate complex relationships between influencing factors (Lee H.-S. et al., 2013). But the traditional means suffers from the one-sidedness and limitations of a single expert’s opinion. There is heterogeneity among experts in terms of professional experience, educational backgrounds, and value preferences; and the judgment results are frequently different, requiring the formation of reasonable weights for expert judgments. The concept lattice technique, actually a concept hierarchical clustering process that essentially describes the association of objects and attributes and integrates the connotations and extensions between concepts, can effectively obtain the expert weighting ratio (Xiao and Zhang, 2020). The CL-WG DEMATEL method could cross validates the results of Study 1 and further distinguishes the importance of the influencing factors, which is an effective and complementary new theoretical exploration tool (Zhang et al., 2022).

### Participants and procedure

The optimized method is described below.

Step 1: Form the muster of impact factors *X* = {*X_1_, X_2,_ …, X_n_*}.

Step 2: The comprehensive evaluation forms the direct relationships between the influencing factors. The initialized direct influence matrix is:


$$z=\left[\begin{array}{ccc} 0 & Z_{12} & \cdots \;\;Z_{1J}\\ Z_{21} & 0 & \begin{array}{cc} \cdots & Z_{2J} \end{array}\\ \begin{array}{c} \cdots \\ Z_{i1} \end{array} & \begin{array}{c} \cdots \\ Z_{i2} \end{array} & \begin{array}{c} \begin{array}{cc} 0 & \cdots \end{array}\\ \begin{array}{cc} \cdots & 0 \end{array} \end{array} \end{array}\right]$$

Step 3: Calculate the new direct influence matrix. Industry or university experts in organizational behavior were invited to score. The evaluation results of a certain class of factors are clustered according to *p* experts. These results are copolymerized into *φ* classes. σ_1_, σ_2_, … σ_φ_ are used to denote the first, second … and nth type; and the same weight is given to experts in the same category. The hypothesis is as follows: *b=*$\overset{p}{\underset{i-1}{\sum }}\sigma_{i}^{2}$, where *g_i_* is the expert weight, $\overset{p}{\underset{i-1}{\sum }}g_{i}\sigma_{i}^{2}$=1, and σ*_i=_bg_i_*. According to the expert weights, the influence coefficient among the factors is obtained as follows: $z_{ij}^{\prime }=\overset{p}{\underset{i-1}{\sum }}g_{i}z_{ij}\left(i,j=1,2,\cdots ,n\right)$. Accordingly, the direct impact matrix after being transformed by the weighting factor is:


$$z=\left[\begin{array}{ccc} 0 & Z_{12}^{\prime } & \begin{array}{cc} \cdots & Z_{1J}^{\prime } \end{array}\\ Z_{21}^{\prime } & 0 & \begin{array}{cc} \cdots & Z_{2J}^{\prime } \end{array}\\ \begin{array}{c} \cdots \\ Z_{i1}^{\prime } \end{array} & \begin{array}{c} \cdots \\ Z_{i2}^{\prime } \end{array} & \begin{array}{c} \begin{array}{cc} 0 & \cdots \end{array}\\ \begin{array}{cc} \cdots & 0 \end{array} \end{array} \end{array}\right]$$

Step 4: Determine the comprehensive impact matrix *T*.

If $g=1/\max\left(\overset{n}{\underset{j=1}{\sum }}Z_{ij}^{\prime }\right)$, where *N = gZ*,Then T $=\underset{k\to \infty }{\lim}\left(N+N^{2}+\cdots N^{K}\right)=N{\left(1-N\right)}^{-1}$ Step 5: According to the integrated impact matrix, calculate the degree of influence, degree affected, degree of centrality, and degree of causation.

The degree of influence (ID): $f_{i}=\overset{n}{\underset{i=1}{\sum }}\;t_{ij}\left(i=1,2,\cdots ,n\right)$

Degree affected (AD): $e_{i}=\overset{n}{\underset{j=1}{\sum }}\;t_{ji}\left(i=1,2,\cdots ,n\right)$

Degree of centrality (CTD): $r_{i}=f_{i}+e_{i}$

Degree of causation (CSD): $z_{i}=f_{i}-e_{i}$

CTD represents the importance in the set of influencing factors, and CSD represents the correlation with other factors. If *Z_i_* is greater than zero, this factor can influence other factors to a greater extent; and if it is less than zero, this factor is more influenced by other factors (Zhang et al., 2022).

### Analysis

According to the steps presented above, the specific data are analyzed as follows.

Step 1: The set of impact factors about MTDC was formed: *X* = {*X_1_, X_2,_ …, X_n_*}.

Step 2: Six experts in organizational behavior were requested, to judge the direct relationships between the influencing factors. The weights of the judgment include 0 = none, 1 = weak … and 5 = strong. The partial initialized direct impact matrix is:


$$Z_{1}=\left[\begin{array}{cc} \begin{array}{cc} \begin{array}{cc} 0 & 4\\ 4 & 0 \end{array} & \begin{array}{cc} 4 & 5\\ 4 & 5 \end{array}\\ \begin{array}{cc} 4 & 4\\ 3 & 5 \end{array} & \begin{array}{cc} 0 & 4\\ 4 & 0 \end{array} \end{array} & \begin{array}{cc} \begin{array}{cc} 2 & 3\\ 4 & 5 \end{array} & \begin{array}{cc} 4 & 3\\ 4 & 4 \end{array}\\ \begin{array}{cc} 2 & 4\\ 3 & 3 \end{array} & \begin{array}{cc} 4 & 3\\ 4 & 3 \end{array} \end{array}\\ \begin{array}{cc} \begin{array}{cc} 4 & 4\\ 3 & 4 \end{array} & \begin{array}{cc} 4 & 5\\ 3 & 4 \end{array}\\ \begin{array}{cc} 4 & 4\\ 4 & 4 \end{array} & \begin{array}{cc} 5 & 4\\ 4 & 2 \end{array} \end{array} & \begin{array}{cc} \begin{array}{cc} 0 & 3\\ 4 & 0 \end{array} & \begin{array}{cc} 4 & 3\\ 4 & 3 \end{array}\\ \begin{array}{cc} 5 & 5\\ 4 & 3 \end{array} & \begin{array}{cc} 0 & 5\\ 5 & 0 \end{array} \end{array} \end{array}\right]$$

$$Z_{2}=\left[\begin{array}{cc} \begin{array}{cc} \begin{array}{cc} 0 & 4\\ 5 & 0 \end{array} & \begin{array}{cc} 4 & 5\\ 4 & 5 \end{array}\\ \begin{array}{cc} 4 & 4\\ 4 & 5 \end{array} & \begin{array}{cc} 0 & 4\\ 4 & 0 \end{array} \end{array} & \begin{array}{cc} \begin{array}{cc} 3 & 4\\ 4 & 5 \end{array} & \begin{array}{cc} 4 & 4\\ 4 & 4 \end{array}\\ \begin{array}{cc} 3 & 4\\ 3 & 3 \end{array} & \begin{array}{cc} 5 & 4\\ 4 & 3 \end{array} \end{array}\\ \begin{array}{cc} \begin{array}{cc} 3 & 4\\ 4 & 4 \end{array} & \begin{array}{cc} 4 & 5\\ 3 & 4 \end{array}\\ \begin{array}{cc} 5 & 4\\ 3 & 4 \end{array} & \begin{array}{cc} 4 & 5\\ 4 & 3 \end{array} \end{array} & \begin{array}{cc} \begin{array}{cc} 0 & 3\\ 4 & 0 \end{array} & \begin{array}{cc} 3 & 4\\ 4 & 3 \end{array}\\ \begin{array}{cc} 5 & 5\\ 4 & 3 \end{array} & \begin{array}{cc} 0 & 4\\ 3 & 0 \end{array} \end{array} \end{array}\right]$$

$$Z_{3}=\left[\begin{array}{cc} \begin{array}{cc} \begin{array}{cc} 0 & 3\\ 5 & 0 \end{array} & \begin{array}{cc} 4 & 4\\ 4 & 4 \end{array}\\ \begin{array}{cc} 5 & 2\\ 2 & 2 \end{array} & \begin{array}{cc} 0 & 5\\ 2 & 0 \end{array} \end{array} & \begin{array}{cc} \begin{array}{cc} 2 & 3\\ 2 & 5 \end{array} & \begin{array}{cc} 4 & 4\\ 4 & 3 \end{array}\\ \begin{array}{cc} 2 & 2\\ 3 & 2 \end{array} & \begin{array}{cc} 4 & 3\\ 4 & 5 \end{array} \end{array}\\ \begin{array}{cc} \begin{array}{cc} 4 & 3\\ 1 & 2 \end{array} & \begin{array}{cc} 2 & 5\\ 2 & 3 \end{array}\\ \begin{array}{cc} 4 & 3\\ 3 & 4 \end{array} & \begin{array}{cc} 4 & 4\\ 4 & 4 \end{array} \end{array} & \begin{array}{cc} \begin{array}{cc} 0 & 4\\ 2 & 0 \end{array} & \begin{array}{cc} 3 & 4\\ 2 & 2 \end{array}\\ \begin{array}{cc} 5 & 4\\ 5 & 3 \end{array} & \begin{array}{cc} 0 & 5\\ 4 & 0 \end{array} \end{array} \end{array}\right]$$

Step 3: Determine the new direct relationship matrix. For example, experts score the relationship between “digital cognition and emotion” and “leadership competency.” According to the clustering results, if the first category is experts 1, 2, 4 and 6 and the second category is experts 3 and 5, then σ_1_ = 4, σ_2_ = 2, *g_1_* = *g_2_* = *g_4_* = *g_6_* =$\frac{4}{20}$, *g_3_* = *g_5_* = $\frac{2}{20}$, and $Z_{12}^{\prime }=4\times \frac{4}{20}+4\times \frac{4}{20}+3\times \frac{2}{20}+4\times \frac{4}{20}+3\times \frac{2}{20}+\frac{4}{20}=3.8$.The calculations of the other influencing factor relationship values are similar. The new direct relationship matrix is as:


$$Z=\left[\begin{array}{llllllll} 0 & 3.8 & 4.0 & 4.2 & 2.5 & 3.5 & 4.04 & 3.8\\ 4.5 & 0 & 4 & 4.21 & 2.64 & 4.21 & 4 & 3.5\\ 4.57 & 3.5 & 0 & 4.04 & 2.8 & 3.57 & 4 & 2.33\\ 3.83 & 3.8 & 3.92 & 0 & 3 & 2.2 & 3.8 & 3.06\\ 3.5 & 3.5 & 3.21 & 4.5 & 0 & 3.5 & 3 & 3\\ 2.5 & 3.36 & 2.8 & 3.5 & 3.21 & 0 & 3.21 & 3\\ 3.79 & 3.21 & 3.79 & 4 & 4.5 & 4 & 0 & 4.21\\ 3.43 & 4 & 4 & 3.36 & 3.79 & 3.5 & 4 & 0 \end{array}\right]$$

Step 4: Calculate the normalized direct relationship matrix *N* (g=1/27.5).


$$N=\left[\begin{array}{cc} \begin{array}{cc} \begin{array}{cc} 0 & 0.1382\\ 0.1636 & 0 \end{array} & \begin{array}{cc} 0.1527 & 0.1527\\ 0.1455 & 0.1532 \end{array}\\ \begin{array}{cc} 0.1662 & 0.1273\\ 0.1394 & 0.1382 \end{array} & \begin{array}{cc} 0 & 0.1469\\ 0.1427 & 0 \end{array} \end{array} & \begin{array}{cc} \begin{array}{cc} 0.0909 & 0.1273\\ 0.0961 & 0.1532 \end{array} & \begin{array}{cc} 0.1469 & 0.1382\\ 0.1455 & 0.1273 \end{array}\\ \begin{array}{cc} 0.1018 & 0.1299\\ 0.1091 & 0.0800 \end{array} & \begin{array}{cc} 0.1455 & 0.0848\\ 0.1382 & 0.1111 \end{array} \end{array}\\ \begin{array}{cc} \begin{array}{cc} 0.1273 & 0.1273\\ 0.0909 & 0.1221 \end{array} & \begin{array}{cc} 0.1169 & 0.1636\\ 0.1018 & 0.1273 \end{array}\\ \begin{array}{cc} 0.1377 & 0.1169\\ 0.1247 & 0.1455 \end{array} & \begin{array}{cc} 0.1377 & 0.1455\\ 0.1455 & 0.1221 \end{array} \end{array} & \begin{array}{cc} \begin{array}{cc} 0 & 0.1273\\ 0.1169 & 0 \end{array} & \begin{array}{cc} 0.1091 & 0.1091\\ 0.1169 & 0.0909 \end{array}\\ \begin{array}{cc} 0.1636 & 0.1455\\ 0.1377 & 0.1273 \end{array} & \begin{array}{cc} 0 & 0.1532\\ 0.1455 & 0 \end{array} \end{array} \end{array}\right]$$

And the integrated impact matrix *T* is:


$$T=\left[\begin{array}{cc} \begin{array}{cc} \begin{array}{cc} 1.2778 & 1.3523\\ 1.4589 & 1.2701 \end{array} & \begin{array}{cc} 1.4006 & 1.4773\\ 1.4351 & 1.5208 \end{array}\\ \begin{array}{cc} 1.3654 & 1.2901\\ 1.3023 & 1.2568 \end{array} & \begin{array}{cc} 1.2128 & 1.4156\\ 1.2951 & 1.2415 \end{array} \end{array} & \begin{array}{cc} \begin{array}{cc} 1.1932 & 1.3100\\ 1.2322 & 1.3685 \end{array} & \begin{array}{cc} 1.4018 & 1.2339\\ 1.4411 & 1.2609 \end{array}\\ \begin{array}{cc} 1.1528 & 1.2601\\ 1.1208 & 1.1805 \end{array} & \begin{array}{cc} 1.3451 & 1.1444\\ 1.2964 & 1.1271 \end{array} \end{array}\\ \begin{array}{cc} \begin{array}{cc} 1.3033 & 1.2605\\ 1.1385 & 1.1247 \end{array} & \begin{array}{cc} 1.2861 & 1.3954\\ 1.1385 & 1.2238 \end{array}\\ \begin{array}{cc} 1.4533 & 1.3900\\ 1.3895 & 1.3587 \end{array} & \begin{array}{cc} 1.4434 & 1.5315\\ 1.3952 & 1.4555 \end{array} \end{array} & \begin{array}{cc} \begin{array}{cc} 1.0328 & 1.2278\\ 1.0204 & 0.9884 \end{array} & \begin{array}{cc} 1.2850 & 1.1350\\ 1.1550 & 1.0021 \end{array}\\ \begin{array}{cc} 1.2998 & 1.3767\\ 1.2313 & 1.3121 \end{array} & \begin{array}{cc} 1.3281 & 1.2941\\ 1.4008 & 1.1133 \end{array} \end{array} \end{array}\right]$$

### Results

According to the integrated impact matrix, calculate the degree of influence, degree affected, degree of centrality and degree of causation. The specific results for each degree show in Table 4, sorted by CTD.

**Table 4:** The results of each degree in the transition phase.

Influence Factor	ID	AD	CTD	CSD	Order
Digital Cognition and Emotion	10.6471	10.6890	21.3360	−0.0419	2
Leadership Competency	10.9877	10.3032	21.2909	0.6845	3
Team Conflict	10.1864	10.6069	20.7933	−0.4205	5
Team Psychological Empowerment	9.8205	11.2615	21.0820	−1.4409	4
Multiteam Structure	9.9258	9.2832	19.2090	0.6426	7
Multiteam Orientation	8.7914	10.0243	18.8156	−1.2329	8
Organizational Digital Climate	11.1168	10.6534	21.7701	0.4634	1
Digital Resource Matching	10.6565	9.3109	19.9674	1.3457	6

Referring to the ranking of CTD, this paper finds that the individual digital cognition and emotion, leadership competence, team psychological empowerment, and organizational digital climate are the preceding influencing factors in the transition phase, and are distributed in three levels. The cross-validation results of study 2 show consistency with study 1.

## Discussion and conclusion

The results of the two studies are consistent, indicating that the cross-validated method has good research reliability. This paper finds that the factors influencing MTDC in the transition phase include multi-level. Our results are similar to one study, which proposed that digital creativity is influenced by multi-level influencing factors, such as individual, team, and the environment (Chamakiotis et al., 2013). Creativity in virtual teams is also similarly influenced by multi-level factors (Ocker, 2005). This indicates that our findings are consistent with the factors influencing creativity (Woodman et al., 1993). However, MTDC has its own uniqueness. MTDC highlights the multiteam level, which is consistent with the prevalence of multi-team working patterns in Internet companies (Zhang et al., 2022).

Further analysis through Study 2 reveals that digital cognition and emotion at the individual level, leadership competency at the individual level, team psychological empowerment at the team level, and organizational digital climate at the organizational level are particularly important for MTDC in the transition phase. The difference in the centrality of MTDC in the transition phase is shown in Table 5. However, this study showed that the importance of multiteam-level influencing factors came low. This may be that the transition phase of multiteam systems is in the formation period, they do not know each other or have no consensus, and need to go through further familiarization and bonding (Xiao and Zhang, 2020); therefore, it is difficult to quickly form digital creativity at the multiteam level, which at this time mainly relies on other levels of influence.

**Table 5:** Difference in the centrality of MTDC in the transition phase.

Influence factors		Action phase
Individual	Digital Cognition and Emotion	✹✹✹✹
	Leadership Competency	✹✹✹
Team	Team Conflict	✹✹
	Team Psychological Empowerment	✹✹✹
Multiteam	Multiteam Structure	✹
	Multiteam Orientation	✹
Organization	Organizational Digital Climate	✹✹✹✹
	Digital Resource Matching	✹✹

The number “✹” indicates the centrality ranking of the influencing factors. The more “✹” the higher the ranking.

The results of the study show that organizational digital climate is the maximum degree of centrality in the influencing factors. MTDC relies heavily on the organizational support climate and digital innovation stimulation. Conservative and entrenched organizations are often too hesitant and worried to adapt to the dynamics and complexity of the digital environment. In the digital environment, organizational climate plays an important role in creativity (Jin et al., 2022). A good innovation support climate can provide a solid organizational guarantee for multiteam strategy development and goal analysis while digital training and stimulation can provide multiteam collaboration normality and innovation awareness. The literature review also shows that organizational climate is closely related to digital creativity (Lee and Chen, 2015). Thus, organizations should focus a lot on creating a better organizational digital climate.

The second most important is digital cognition and emotion. It means clearly understanding the organization’s digital strategic goals and fully engaging in their work. It suggests that individual cognition and emotion are key to stimulating creativity (Ocker, 2005). E.g., individuals who possess a “promotion focus” (Jin et al., 2016), have good digital skills, and are more inclined to digital trends, will further promote creative activities. Individuals with good digital cognition will be able to fully understand the assignment of tasks in the transition phase and maximize their potential. These individual factors are closely related to digital creativity (Lee and Chen, 2015). Thus, stimulating MTDC is closely related to digital cognition and emotion.

The results of the study indicate that leadership competency ranks third in centrality. Leadership is a core factor in multi-team systems (DeChurch and Marks, 2006; Zaccaro et al., 2020). The coordination of multiteam boundaries is often the responsibility of team leaders, and the ability of individual leaders to act across boundaries is critical to multiteam (de Vries et al., 2022). In addition, leaders with extensive social network resources can provide richer perspectives on multiteam goal setting and task coordination (Zaccaro et al., 2020). Thus, organizations should develop and select leaders who match. Meanwhile, leaders should actively adapt to the needs of the organization.

Finally, the fourth ranking in centrality is team psychological empowerment. This is an intrinsic and continuous work motivator that enhances an organization’s intrinsic level of performance (Rosen, 1999; Maynard et al., 2013). One study confirms a positive correlation between psychological empowerment and organizational creativity (Zhang and Bartol, 2010). An empirical study of a large digital technology company found that empowered leaders positively influence psychological empowerment, which in turn influences intrinsic motivation and creative engagement processes (Zhang and Bartol, 2010). Team psychological empowerment can stimulate team creative processes and thus promote multiteam creativity.

## Implication

This paper adopts an interactive validation manner to explore and analyze the factors influencing MTDC of high-tech firms. Study 1 adopts a qualitative analysis method such as the rooting theory to fully explore the influencing factors, and study 2 uses the CL-WG DEMATEL method to further verify and analyze the importance of factors. The two cross-validated research methods further enrich the methodology in the field of the organization.

Second, this paper constructs a theoretical framework of MTDC influencing factors in the transition phase. This study indicates that influencing factors of MTDC are multi-levels, consisting of individual, team, multiteam, and organizational levels. In the transition phase, the individual level comprises digital cognition and emotion, and leadership competency, the team level comprises psychological empowerment, and team conflict, the multiteam level comprises the multiteam structure, and the multiteam orientation, the organizational level comprises the organizational digital climate, and digital resource matching. This study responds to the theoretical call of further exploring MTDC and digital creativity (Shao et al., 2021; Zhang et al., 2022), and this paper further enriches the theory of digital creativity and MTDC. Meanwhile, this paper provides a practical reference on how to stimulate MTDC in organizations, fully stimulating MTDC is a multi-level system engineering.

Third, further analysis of the results of Study 2 shows that the influencing factors of MTDC have differential centrality. The organizational digital climate, team psychological empowerment, individual digital cognition and emotion, and leadership competency are particularly important for MTDC during the transition phase. As it is obvious, the transition phase has its uniqueness. Thus, organizations should pay more attention to partial influencing factors during the transition phase, especially those with high centrality. This paper provides a theoretical and practical reference for the organization to focus on which critical factors in the transition phase. In the process of implementation, the organization should take different measures according to their importance in the case of limited resources.

Finally, the paper finds both differences and similarities between the influencing factors of MTDC and organizational creativity. Although MTDC is a study of creativity, MTDC focuses more on the digital domain (Zhang et al., 2022), such as digital cognition, digital structure, digital innovation culture, digital human capital, platform and ecological resources, and so on. Also, the influencing factors of MTDC include not only individual, team, and organizational levels, but also multiteam level, such as multiteam structure and multiteam orientation. This is not consistent with literature studies on the factors influencing creativity (Woodman et al., 1993), which do not focus on the multiteam level. Thus, in the digital era where multi-team working patterns are increasingly prevalent, organizations should both follow the rules of digital development and conform to the inherent mechanisms of MTS (Zhang et al., 2022).

This study responds to the call for further research from the MTS perspective (Lei et al., 2022; Wu et al., 2022) and the transition phase perspective (Zhang et al., 2022), and constructs a specific and systematic frame of the influencing factors of MTDC specifically for the transition phase, which provides a theoretical and practical reference for organizations to conduct precise scientific and rational management and achieve efficient and dynamic creative development.

## Limitations

This paper has some limitations. First, the sample size of this study is relatively small. However, this paper adopts the cross-validation method to justify the results, and the conclusions of the two studies are consistent, which shows that the research results are effective. Previous studies have similarly shown that such a sample size is appropriate in studies like the two cross-validation research approaches (Shi et al., 2016; Xiao and Zhang, 2020; Zhang et al., 2022). For future studies, the sample size could be appropriately expanded to enhance the validity of the study. Second, the interviewees are mainly from companies in China’s Yangtze River Delta region. Although the selected companies are at the forefront of digital construction and can better reflect the typical situation of MTDC, the sample is still rather one-sided. For future studies, a wider range of companies and a wider range of countries could be selected to increase the representativeness of the results (Zhang et al., 2022). Third, the results of the interviews are not tested by other various research approaches. This paper adopts the method of grounded theory and the CL-WG DEMATEL method while following the principle of matching theory and data. Future research could adopt more diverse research methods such as the full-cycleresearch approach, or empirical studies with large samples, thus taking advantage of the strengths of various research methods (Chatman and Flynn, 2005).

## Data availability statement

The original contributions presented in the study are included in the article/Supplementary material, further inquiries can be directed to the corresponding author/s.

## Author contributions

WC and JZ: conceptualization, methodology, writing – review and editing, project administration, and funding acquisition. WC, YC, and JZ: software, formal analysis, investigation, data curation, writing – original draft preparation, and visualization. YX: supervision. WC, JZ, and BW: validation. All authors contributed to the article and approved the submitted version.

## Funding

This paper was supported by the Zhejiang Provincial Philosophy and Social Sciences Planning Project (23NDJC031Z), Zhejiang Province Soft Science Research Plan Project of China (2022C35072), Humanity and Social Science Foundation of Ministry of Education of China (21YJCZH213), National Natural Science Foundation of China (72074195), and National Social Science Foundation of China (20CTQ010).

## Conflict of interest

The authors declare that the research was conducted in the absence of any commercial or financial relationships that could be construed as a potential conflict of interest.

## Publisher’s note

All claims expressed in this article are solely those of the authors and do not necessarily represent those of their affiliated organizations, or those of the publisher, the editors and the reviewers. Any product that may be evaluated in this article, or claim that may be made by its manufacturer, is not guaranteed or endorsed by the publisher.

## References

[ref1] Amabile T. M. (1988). A model of creativity and innovation in organizations. Res. Organ. Behav. 10, 123–167.

[ref2] Amabile T. M. Conti R. Coon H. Lazenby J. Herron M. (1996). Assessing the work environment for creativity. Acad. Manag. J. 39, 1154–1184. doi: 10.2307/256995

[ref3] Chamakiotis P. Dekoninck E. A. Panteli N. (2013). Factors influencing creativity in virtual design teams: an interplay between technology, teams and individuals. Creat. Innov. Manag. 22, 265–279. doi: 10.1111/caim.12039

[ref4] Chatman J. A. Flynn F. J. (2005). Full-cycle micro-organizational behavior research. Organ. Sci. 16, 434–447. doi: 10.1287/orsc.1050.0136

[ref5] Chen W. Xiao Y. Liu Y. Wang B. (2022). The relationship of employees’ promotion focus and job crafting: psychological empowerment as a mediator. Soc. Behav. Personal. Int. J. 50:e11467, 20–29. doi: 10.2224/sbp.11467

[ref6] Cuijpers M. Uitdewilligen S. Guenter H. (2016). Effects of dual identification and interteam conflict on multiteam system performance. J. Occup. Organ. Psychol. 89, 141–171. doi: 10.1111/joop.12113

[ref7] Davison R. B. Hollenbeck J. R. Barnes C. M. Sleesman D. J. Ilgen D. R. (2012). Coordinated action in multiteam systems. J. Appl. Psychol. 97, 808–824. doi: 10.1037/a0026682, PMID: 22201246

[ref8] De Vries T. A. Hollenbeck J. R. Davison R. B. Walter F. Van der Vegt G. S. (2016). Managing coordination in multiteam systems: integrating micro and macro perspectives. Acad. Manag. J. 59, 1823–1844. doi: 10.5465/amj.2014.0385

[ref9] de Vries T. A. van der Vegt G. S. Bunderson J. S. Walter F. Essens P. J. M. D. (2022). Managing boundaries in multiteam structures: from parochialism to integrated pluralism. Organ. Sci. 33, 311–331. doi: 10.1287/orsc.2021.1436

[ref10] DeChurch L. A. Marks M. A. (2006). Leadership in multiteam systems. J. Appl. Psychol. 91, 311–329. doi: 10.1037/0021-9010.91.2.311, PMID: 16551186

[ref11] Glaser B. G. (1978) in Theoretical sensitivity. ed. Valley M. (CA: The Sociology Press)

[ref12] Grover P. Kar A. K. Dwivedi Y. K. (2022). Understanding artificial intelligence adoption in operations management: insights from the review of academic literature and social media discussions. Ann. Oper. Res. 308, 177–213. doi: 10.1007/s10479-020-03683-9

[ref13] Hahm S. (2017). Information sharing and creativity in a virtual team: roles of authentic leadership, sharing team climate and psychological empowerment. KSII Trans. Internet Inf. Syst. 11, 4105–4119. doi: 10.3837/tiis.2017.08.020

[ref14] Hoda R. Noble J. Marshall S. (2012). Developing a grounded theory to explain the practices of self-organizing agile teams. Empir. Softw. Eng. 17, 609–639. doi: 10.1007/s10664-011-9161-0

[ref15] Jehn K. A. Mannix E. A. (2001). The dynamic nature of conflict: a longitudi-nal study of intragroup conflict and group performance. Acad. Manag. J. 44, 238–251. doi: 10.2307/3069453

[ref16] Jin S. Li Y. Xiao S. (2022). What drives Employees' innovative behaviors in emerging-market multinationals? An integrated approach. Front. Psychol. 12:803681. doi: 10.3389/fpsyg.2021.803681, PMID: 35126255 PMC8810652

[ref17] Jin X. Wang L. Dong H. (2016). The relationship between self-construal and creativity - regulatory focus as moderator. Pers. Individ. Differ. 97, 282–288. doi: 10.1016/j.paid.2016.03.044

[ref18] Lanaj K. Hollenbeck J. R. Ilgen D. R. Barnes C. M. Harmon S. J. (2013). The double-edged sword of decentralized planning in multiteam systems. Acad. Manag. J. 56, 735–757. doi: 10.5465/amj.2011.0350

[ref19] Lee M. R. Chen T. T. (2015). Digital creativity: research themes and framework. Comput. Hum. Behav. 42, 12–19. doi: 10.1016/j.chb.2014.04.001

[ref20] Lee D. S. Lee K. C. Jo N. Y. (2013) in Digital creativity: Individuals, groups, and organizations. ed. Lee K. C. (New York: Springer)

[ref21] Lee H.-S. Tzeng G.-H. Yeih W. Wang Y.-J. Yang S.-C. (2013). Revised DEMATEL: resolving the infeasibility of DEMATEL. Appl. Math. Model. 37, 6746–6757. doi: 10.1016/j.apm.2013.01.016

[ref22] Lei Y. Wu X. Jiang J. (2022). Shared mental models and boundary management activities in new product development: a perspective on multiteam systems. IEEE Trans. Eng. Manag. 7, 1–13. doi: 10.1109/tem.2022.3147905

[ref23] Luciano M. M. DeChurch L. A. Mathieu J. E. (2018). Multiteam systems: a structural framework and meso-theory of system functioning. J. Manag. 44, 1065–1096. doi: 10.1177/0149206315601184

[ref24] Luciano M. M. Nahrgang J. D. Shropshire C. (2020). Strategic leadership systems: viewing top management teams and boards of directors from a multiteam systems perspective. Acad. Manag. Rev. 45, 675–701. doi: 10.5465/amr.2017.0485

[ref25] Marks E. Mathieu J. E. Zaccaro S. J. (2001). A temporally based framework and taxonomy of team processes. Acad. Manag. Rev. 26, 356–376. doi: 10.2307/259182

[ref26] Mathieu J. E. Marks M. A. Zaccaro S. J. (2001). “Multi-team systems,” in International Handbook Of Work And Organizational Psychology. eds Anderson N. Ones D. Sinangil H. K. Viswesvaran C. (London: Sage), 289–313.

[ref27] Mathieu J. E. Schulze W. (2006). The influence of team knowledge and formal plans on episodic team process-performance relationships. Acad. Manag. J. 49, 605–619. doi: 10.5465/amj.2006.21794678

[ref28] Maynard M. T. Mathieu J. E. Gilson L. L. O'Boyle E. H. Cigularov K. P. (2013). Drivers and outcomes of team psychological empowerment: a meta-analytic review and model test. Organ. Psychol. Rev. 3, 101–137. doi: 10.1177/2041386612456868

[ref29] Mell J. N. DeChurch L. A. Leenders R. T. A. J. Contractor N. (2020). Identity asymmetries: an experimental investigation of social identity and information exchange in multiteam systems. Acad. Manag. J. 63, 1561–1590. doi: 10.5465/amj.2018.0325

[ref30] Mello J. Flint D. J. (2009). A refined view of grounded theory and its application to logistics research. J. Bus. Logist. 30, 107–125. doi: 10.1002/j.2158-1592.2009.tb00101.x

[ref31] Ocker R. J. (2005). Influences on creativity in asynchronous virtual teams: a qualitative analysis of experimental teams. IEEE Trans. Prof. Commun. 48, 22–39. doi: 10.1109/TPC.2004.843294

[ref32] Pérez-Fuentes M. D. C. Molero Jurado M. Oropesa Ruiz N. F. Simón Márquez M. D. M. Gázquez Linares J. J. (2019). Relationship between digital creativity, parenting style, and adolescent performance. Front. Psychol. 10:2487. doi: 10.3389/fpsyg.2019.02487, PMID: 31787913 PMC6853992

[ref33] Porck J. P. Matta F. K. Hollenbeck J. R. Oh J. K. Lanaj K. Lee S. M. (2019). Social identification in multiteam systems: the role of depletion and task complexity. Acad. Manag. J. 62, 1137–1162. doi: 10.5465/amj.2017.0466

[ref34] Rapp T. L. Mathieu J. E. (2019). Team and individual influences on members' identification and performance per membership in multiple team membership arrangements. J. Appl. Psychol. 104, 303–320. doi: 10.1037/apl0000344, PMID: 30091620

[ref35] Rosen K. B. (1999). Beyond self-management: antecedents and consequences of team empowerment. Acad. Manag. J. 42, 58–74. doi: 10.2307/256874

[ref36] Runco M. A. Kim D. (2013). Four Ps of creativity and recent updates. New York: Springer.

[ref37] Shao Z. Li X. Wang Q. (2021). From ambidextrous learning to digital creativity: an integrative theoretical framework. Inf. Syst. J. 32, 544–572. doi: 10.1111/isj.12361

[ref38] Shi L. P. Jia Y. N. Liu Q. (2016). Exploratory research into influence factors of team goal orientation: based on the methods of grounded theory and concept lattice-weighted group dematel. Oper. Res. Manag. Sci. 25, 104–112. doi: 10.12005/orms.2016.0051

[ref39] Torres E. M. Wallace D. M. Zaccaro S. J. Dubrow S. (2021). Deconstructing multiteam system action: development and content validation of a multilevel multiteam system action taxonomy. Hum. Perform. 34, 189–216. doi: 10.1080/08959285.2021.1922909

[ref40] Urquhart C. Lehmann H. Myers M. D. (2010). Putting the 'theory' back into grounded theory: guidelines for grounded theory studies in information systems. Inf. Syst. J. 20, 357–381. doi: 10.1111/j.1365-2575.2009.00328.x

[ref41] van de Brake H. J. Walter F. Rink F. A. Essens P. J. M. D. van der Vegt G. S. (2020). Benefits and disadvantages of individuals' multiple team membership: the moderating role of organizational tenure. J. Manag. Stud. 57, 1502–1530. doi: 10.1111/joms.12539

[ref42] Van den Berg W. Curseu P. L. Meeus M. T. H. (2014). Emotion regulation and conflict transformation in multi-team systems. Int. J. Confl. Manag. 25, 171–188. doi: 10.1108/ijcma-05-2012-0038

[ref43] Van Rensburg C. J. Coetzee S. A. Schmulian A. (2021). Developing digital creativity through authentic assessment. Assess. Eval. High. Educ. 47, 857–877. doi: 10.1080/02602938.2021.1968791

[ref44] Wijnmaalen J. Voordijk H. Rietjens S. Dewulf G. (2019). Intergroup behavior in military multiteam systems. Hum. Relat. 72, 1081–1104. doi: 10.1177/0018726718783828

[ref45] Woodman R. W. Sawyer J. E. Griffin R. W. (1993). Toward a theory of organizational creativity. Acad. Manag. Rev. 18, 293–321. doi: 10.5465/AMR.1993.3997517

[ref46] Wu J. Richard O. C. Triana M. Zhang X. (2022). The performance impact of gender diversity in the top management team and board of directors: a multiteam systems approach. Hum. Resour. Manag. 61, 157–180. doi: 10.1002/hrm.22086

[ref47] Xiao Y. Zhang Y. (2020). Identifying critical factors in multi-team cohesion from the perspective of performance episode: empirical evidence from Chinese manufacturers. J. Bus. Econ. 339, 27–39. doi: 10.14134/j.cnki.cn33-1336/f.2020.01.003

[ref48] Yoo Y. J. Henfridsson O. Lyytinen K. (2010). The new organizing logic of digital innovation: an agenda for information systems research. Inf. Syst. Res. 21, 724–735. doi: 10.1287/isre.1100.0322

[ref49] Zaccaro S. J. Dubrow S. Torres E. M. Campbell L. N. P. (2020). Multiteam systems: an integrated review and comparison of different forms. Annu. Rev. Organ. Psych. Organ. Behav. 7, 479–503. doi: 10.1146/annurev-orgpsych-012119-045418

[ref50] Zhang X. Bartol K. M. (2010). Linking empowering leadership and employee creativity: the influence of psychological empowerment, intrinsic motivation, and creative process engagement. Acad. Manag. J. 53, 107–128. doi: 10.5465/amj.2010.48037118

[ref51] Zhang J. Chen W. L. Xiao Y. C. Wang B. H. (2022). Exploration of digital creativity: construction of the multiteam digital creativity influencing factor model in the action phase. Front. Psychol. 13:822649. doi: 10.3389/fpsyg.2022.822649, PMID: 35651558 PMC9150790

